# Hierarchically Converged Defect Engineering with 2-Dimensional Black Phosphorus/MXene Sequence for Sensitive Photoelectrochemical-Electrostatic Sensors

**DOI:** 10.34133/research.0966

**Published:** 2025-10-24

**Authors:** Wei Zeng, Yuan Zhang, Zhengyin Wu, Zichu Zhang, Liting Deng, Yuan Tian, Mengying Che, Yiming Chen, Yi Xiong, Yumin Wang, Pengfei Fang, Yun Tang, Shuoxue Jin, Suiting Ning

**Affiliations:** ^1^ National Key Laboratory of Opto-Electronic Information Acquisition and Protection Technology, Anhui University, Hefei 230601, Anhui, China.; ^2^ Analytical and Testing Center, School of Bioengineering and Health, School of Electronics and Electrical Engineering, Hubei Provincial Engineering Research Center for Wide-Bandgap Semiconductor Materials and Devices, Wuhan Textile University, Wuhan, 430073, Hubei, China.; ^3^ School of Physics and Technology, Key Laboratory of Nuclear Solid State Physics Hubei Province, Wuhan University, Wuhan 430072, China.; ^4^ Multi-disciplinary Research Division, Institute of High Energy Physics, Chinese Academy of Sciences, Beijing 100049, China.; ^5^ School of Science, Hubei University of Technology, Wuhan 430068, China.

## Abstract

The function-integrated sensors have harvested increasingly vital across diverse applications, especially in the field of smart wearables, yet optimizing the synergy between electrostatic and light signals in photoelectrochemical (PEC) systems remains a critical challenge. Here, we fabricate a hierarchical ZnO/Bi_2_O_3_/BiOCl/black phosphorus (BP)/MXene (Ti_3_C_2_T*_x_*) heterostructure and develop a PEC sensor tailored for electrostatic coupling and surface electromyography (sEMG) detection. Sequential integration of 2-dimensional (2D) BP and Ti_3_C_2_T*_x_* precisely repairs defects in the 3D ZnO/Bi_2_O_3_/BiOCl framework, enhancing electrode contact area and forming multidimensional heterostructures that markedly boost electron–hole separation. Under illumination or ambient electrostatic field (AEF) stimulation, the composite electrode outperforms its pristine counterpart, achieving a photocurrent of 20.46 μA cm^−2^ under 30-W blue light, which is 2.4 times higher than the device without the 2D layers. A synergistic AEF–light effect further enhances carrier transport, amplifying AEF detection sensitivity. These results highlight defect engineering as a robust strategy for advancing PEC performance in PEC applications and enable a light-assisted sEMG sensor with marked signal improvement.

## Introduction

Multifunctional sensors are critical components in electronic devices, enabling real-time health diagnostics, therapeutics, environmental monitoring, and smart sensing applications. Photoelectrochemical (PEC) and electrostatic field sensors show exceptional promise for ultrasensitive DNA detection [1], electromagnetic pulse measurement [2], and bioelectric signal acquisition, such as electroencephalography [3] and electromyography [4]. Surface electromyography (sEMG) sensors, which transduce biopotentials via skin-mounted electrodes, are vital for noninvasive health monitoring. However, their weak signal strength complicates differentiation of exercise intensities [4–7], necessitating enhanced sensitivity through innovative device design and material optimization.

The conventional Ag/AgCl wet electrodes exhibit signal attenuation due to conductive gel dehydration, while rigid, bulky dry electrodes suffer from poor skin contact and delamination during deformation [8,9]. Recent advances demonstrate that the 30-W blue light enhances PEC signals, with WO_3_/Bi_10_O_6_S_9_/black phosphorus (BP) heterojunctions achieving a photocurrent of 12.51 nA cm^−2^ under illumination, which is 2.2 times higher than without illumination (5.52 nA cm^−2^) [10]. Ambient electrostatic field (AEF) further promotes light-induced charge separation and band structure modulation [11], synergizing with light-induced built-in electric fields when aligned with semiconductor energy level gradients [10–13]. For instance, WO_3_/BiVO_4_/MXene (Ti_3_C_2_T*_x_*) heterojunctions yield a photocurrent of 1.39 mA cm^−2^ under combined AEF and light, which is 33.9 times higher than that under AEF alone (0.041 mA cm^−2^). Inspired by the principles of PEC and the synergistic effect of electrostatic fields, the light and electrostatic fields are suggested to enhance sEMG signals.

The silicon (Si), valued for its tunable conductivity, serves as a robust substrate for functional films [14,15]. However, it tends to oxidize under bias or light irradiation in aqueous media [15,16]. Metal oxide coatings such as WO_3_, Bi_2_O_3_, CdO, and TiO_2_ can enhance stability and reduce the Fermi level pinning at the Schottky contacts and offer favorable semiconductor properties [15,17–21]. The rapid carrier recombination, however, degrades Si-based device performance, prompting the use of heterojunctions to accelerate charge transfer [22]. The Bi/BiOCl/Bi_2_O_2_CO_3_ heterojunction achieves 150 nA cm^−2^, which is approximately 2 times higher than that of the electrode without Bi/BiOCl films, via enhanced visible light absorption and plasmonic charge separation [23]. Similarly, ZnO/BP heterojunctions reach 0.6 μA cm^−2^, tripling ZnO photocurrent, due to broadened electron pathways and visible light absorption [24]. The defect engineering, such as repairing nanoscale pores with 2-dimensional (2D) materials, markedly boosts performance [12]. Conductive co-catalysts like Ti_3_C_2_T*_x_* in ZnO/Ti_3_C_2_T*_x_* (37 μA cm^−2^, increase of 4.6 times) form Schottky barriers, curbing recombination and extending absorption [25,26]. While nano-heterojunctions, such as BP/Bi_10_O_6_S_9_, advance bioelectric sensing [10,27], exploiting external fields like light and AEF for sEMG enhancement remains underexplored.

Herein, we report a ZnO/Bi_2_O_3_/BiOCl/BP/MXene (ZnBiPM) photoanode, leveraging AEF and multidimensional heterostructures to enhance PEC-based sEMG sensing. Defect repair within 3D hetero-frameworks, driven by BP and Ti_3_C_2_T*_x_* on a Si-supported ZnO/Bi_2_O_3_/BiOCl layer, combined with the high conductivity of MXene, has significantly improved charge separation and transfer. Photocurrent scales with AEF direction and light intensity, enabling precise signal amplification. This rigorously validated design markedly enhances sEMG sensitivity, offering a transformative platform for bioelectric monitoring with broad implications.

## Results and Discussion

**Fig. 1.**
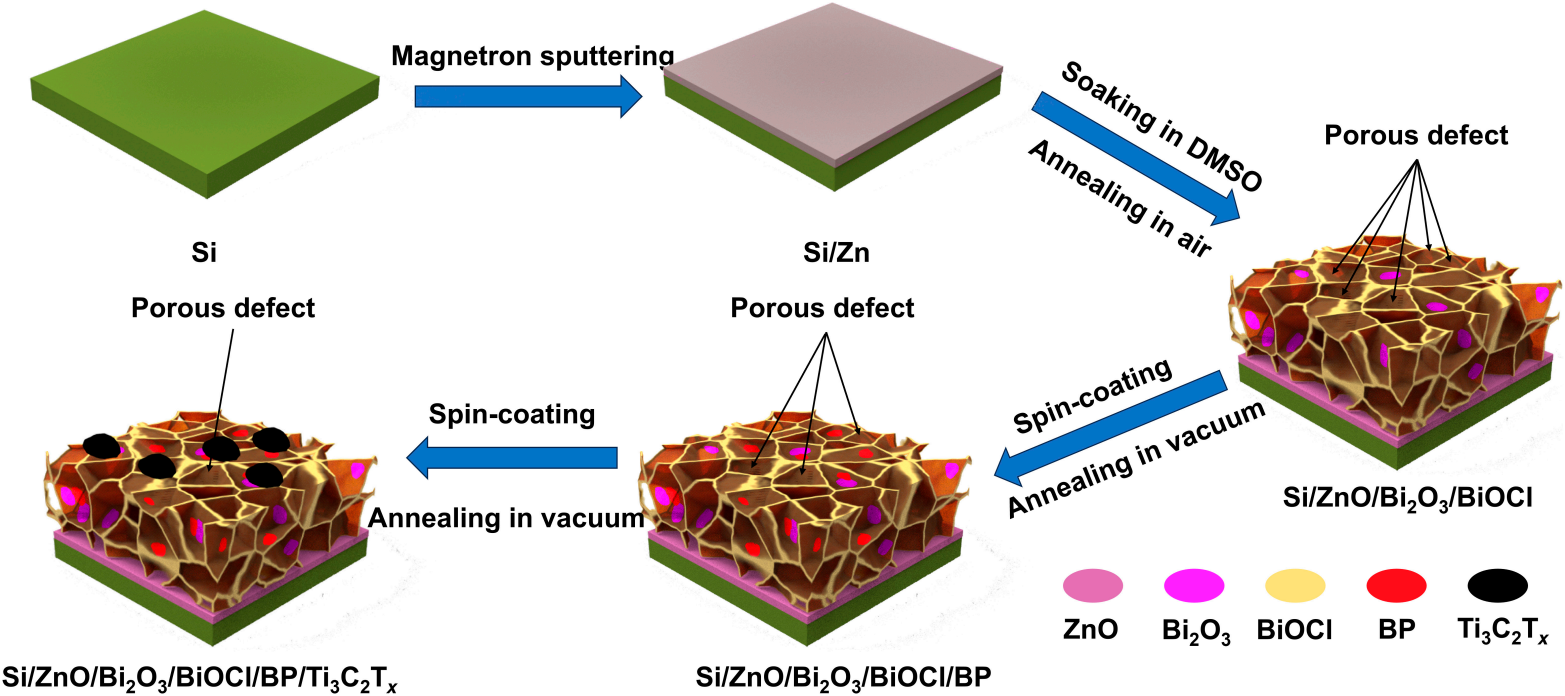
Schematic illustration for fabrication process of ZnBiPM electrode.

The fabrication of the ZnBiPM electrode is illustrated in Fig. 1. Initially, a Si wafer (green substrate) underwent plasma etching under vacuum to remove its native oxide layer, followed by magnetron sputtering of Zn (gray layer) to yield a Si/Zn substrate. Subsequent galvanic replacement and hydrolysis reactions deposited Bi_2_O_3_ (purple layer) and BiOCl (yellow layer), forming a Si/ZnO/Bi_2_O_3_/BiOCl heterostructure with inherent defects within its 3D framework. Annealing oxidized the Zn layer into ZnO (pink layer). Defects in the Si/ZnO/Bi_2_O_3_/BiOCl stack were progressively mitigated through spin-coating and annealing of BP (red layer) nanosheets. Finally, Ti_3_C_2_T*_x_* (black pieces) was spin-coated and vacuum-annealed onto the Si/ZnO/Bi_2_O_3_/BiOCl/BP surface, further repairing defects and yielding the ZnBiPM composite electrode. This stepwise process establishes a robust, defect-engineered heterostructure optimized for PEC performance.

### Morphology and structural characterization

**Fig. 2.**
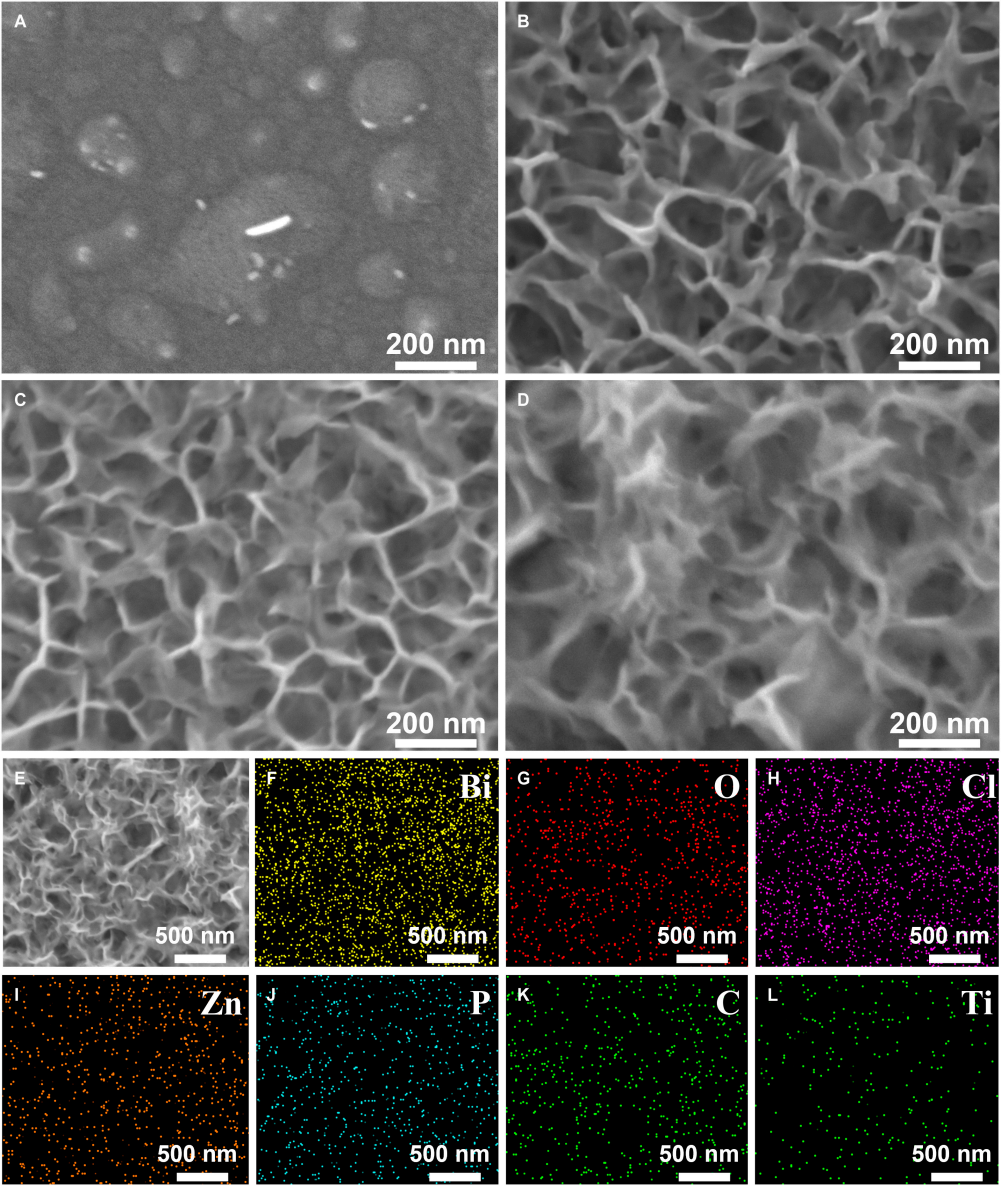
SEM images of (A) Si/Zn, (B) ZnBi, (C) ZnBiP, and (D) ZnBiPM electrodes. (F to L) EDX mapping images of Bi, O, Cl, Zn, P, C, and Ti elements derived from the ZnBiPM electrode on selected area (E).

To elucidate the structural evolution driven by defect engineering, the surface morphology and microstructure are investigated for Si/Zn, ZnO/Bi_2_O_3_/BiOCl (ZnBi), ZnO/Bi_2_O_3_/BiOCl/BP (ZnBiP), and ZnBiPM electrodes using scanning electron microscopy (SEM), as presented in Fig. 2. The Si/Zn substrate (Fig. 2A) exhibits an island-like Zn layer atop the Si surface, with bright spots likely attributable to ZnO, given its reduced conductivity and resultant charge-induced contrast enhancement. For the ZnBi electrode (Fig. 2B), Bi_2_O_3_/BiOCl layers form atop Si/Zn via galvanic replacement, yielding a porous structure within a 3D hetero-framework, with an average pore size of ~195.4 nm. Upon incorporation of BP nanosheets (Fig. 2C), the ZnBiP electrode displays a reduced pore size of ~169 nm, suggesting that spin-coated BP anchors to the underlying framework via chemical bonds [confirmed by subsequent x-ray photoelectron spectroscopy (XPS) analysis], partially filling surface pores and passivating internal defects. Further coating with Ti_3_C_2_T*_x_* (Fig. 2D) yields the ZnBiPM electrode, where the pore diameter shrinks to ~86 nm, indicating effective defect coverage and infilling by MXene within the 3D heterostructure. Cross-sectional SEM images (Fig. S1) corroborate this trend, showing progressive flattening and pore reduction across the series. The energy-dispersive x-ray spectroscopy (EDX) analysis of the ZnBiPM electrode (target area; Fig. 2E) reveals uniform distribution of Bi, O, Cl, Zn, P, C, and Ti (Fig. 2F to L), confirming the successful integration of all components.

**Fig. 3.**
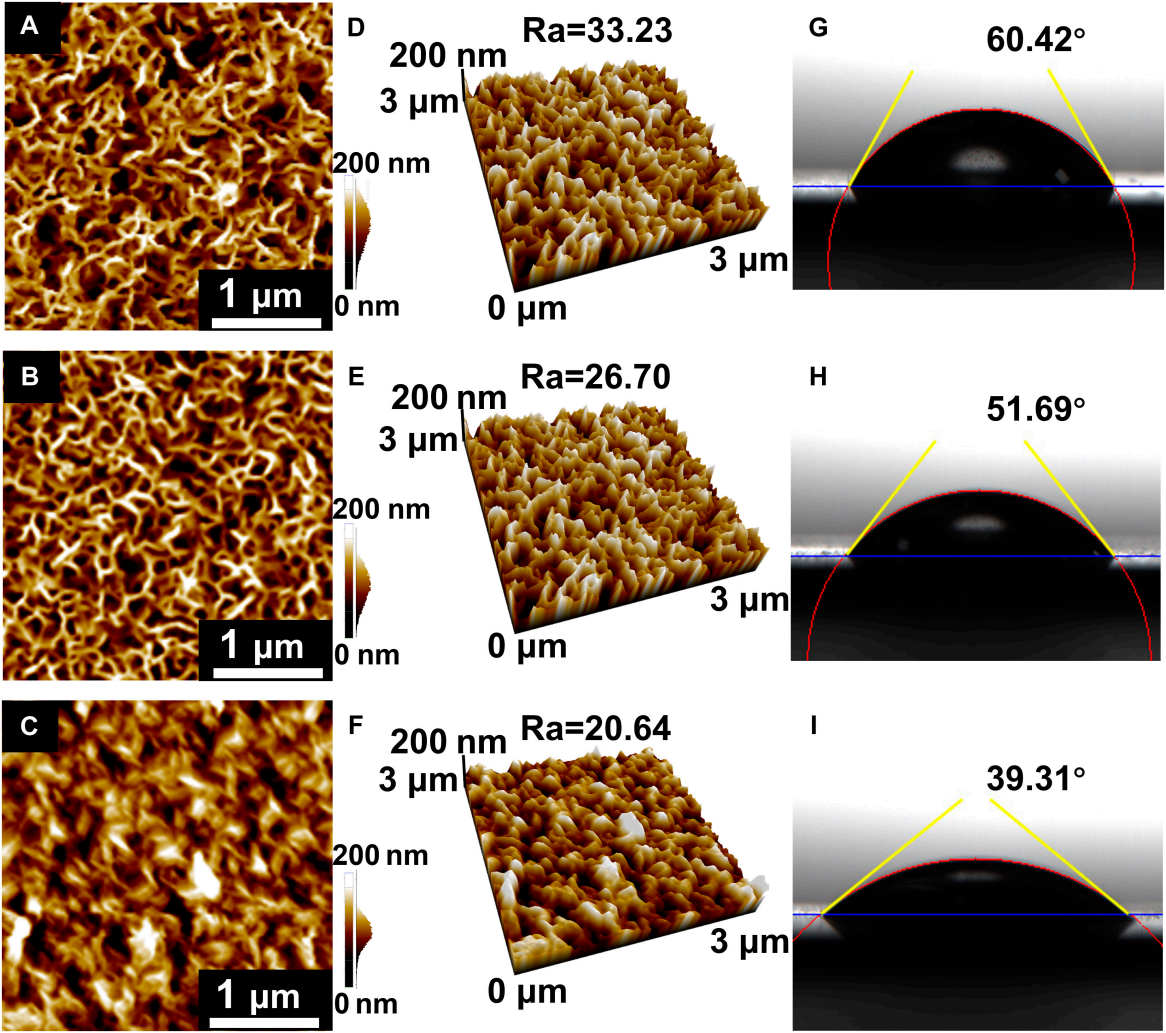
The AFM images of (A) ZnBi, (B) ZnBiP, and (C) ZnBiPM electrodes. The 3D AFM images of (D) ZnBi, (E) ZnBiP, and (F) ZnBiPM electrodes. The contact angle images of (G) ZnBi, (H) ZnBiP, and (I) ZnBiPM electrodes.

**Fig. 4.**
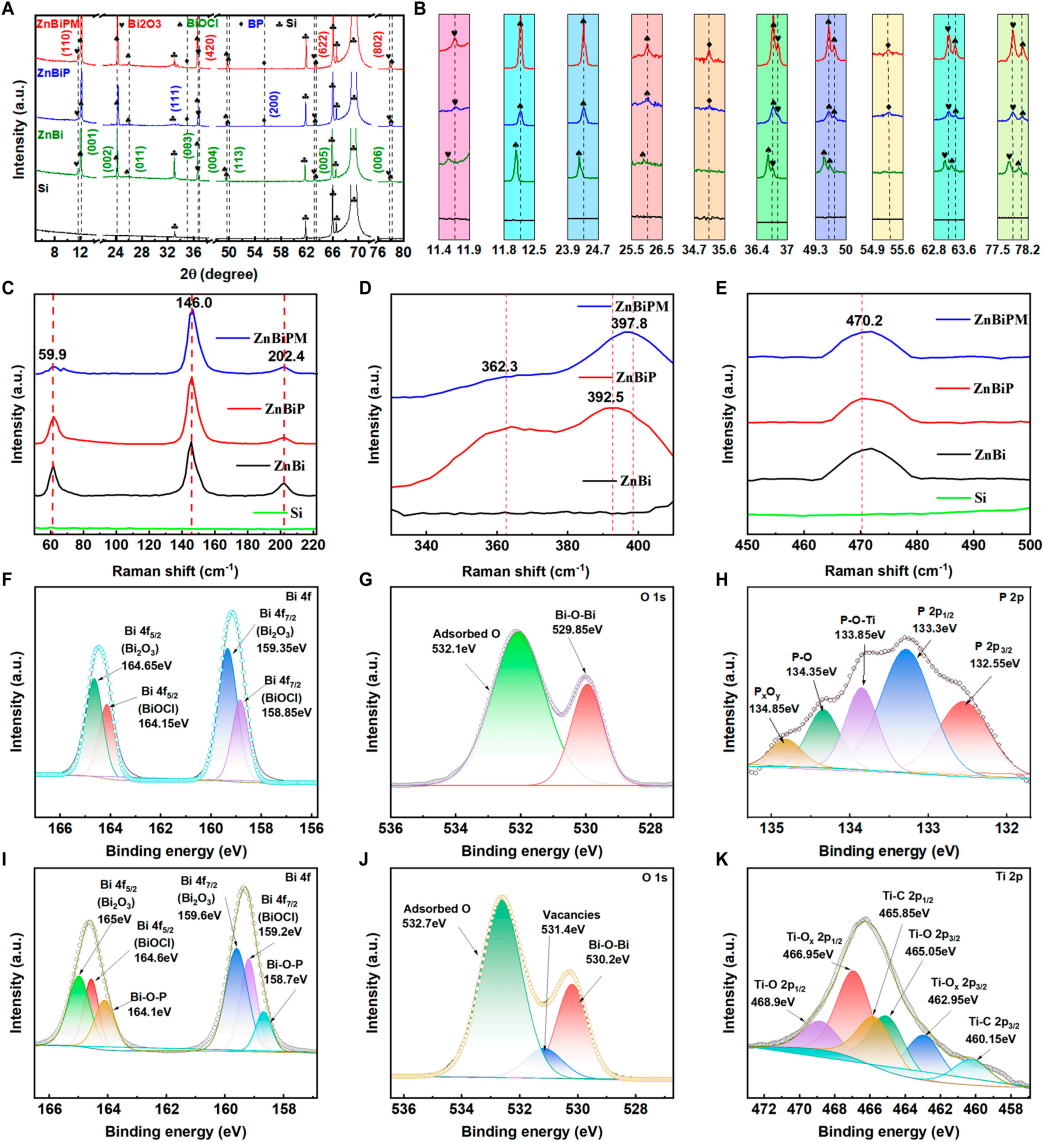
(A) XRD patterns of Si, ZnBi, ZnBiP, and ZnBiPM electrodes. (B) Enlarged local XRD pattern of (A). (C to E) Raman spectrum of ZnBi, ZnBiP, and ZnBiPM electrodes. XPS spectra of the ZnBi electrode: high-resolution XPS spectra of (F) Bi and (G) O. XPS spectra of the ZnBiPM electrode: high-resolution XPS spectra of (H) P, (I) Bi, (J) O, and (K) Ti.

To further evaluate the structural evolution and defect repair promoted by defect engineering, we conducted atomic force microscopy (AFM) and contact angle measurements, and the results are shown in Fig. 3. AFM images of the ZnBi, ZnBiP, and ZnBiPM electrodes are shown in Fig. 3A to F, revealing surface roughness values of 33.23, 26.70, and 20.64 nm, respectively. The progressive decrease in roughness following the addition of BP indicates effective infilling of defects within the 3D hetero-framework of the ZnBi electrode, aligning with SEM observations (Fig. 2B). Incorporation of Ti_3_C_2_T*_x_* further reduces the roughness of the ZnBiPM electrode relative to ZnBiP, evidencing additional defect repair (consistent with Fig. 2C). This trend is corroborated by cross-sectional SEM data (Fig. S1), which show a flattening of the electrode profile. 3D AFM reconstructions (Fig. 3D to F) provide detailed visualization of the hierarchical defect filling by BP and MXene, underscoring their role in smoothing the electrode surface. Through surface analysis experiments using XPS and Raman spectroscopy (as shown in Fig. 4C to K), hydrophilic groups such as -OH and -NH_2_ or hydrophobic groups such as -CH_3_ were not observed. Therefore, the contact angle experimental results in this paper are attributed to the influence of the surface roughness of the electrode structure.

Contact angle measurements (Fig. 3G to I) yield values of 60.42°, 51.69°, and 39.31° for ZnBi, ZnBiP, and ZnBiPM electrodes, respectively. Given that higher contact angles correlate with greater surface roughness [28], these results align quantitatively with AFM and SEM findings. The sequential reduction in contact angle confirms that BP and Ti_3_C_2_T*_x_* progressively fill nanoscale pores, diminishing both surface roughness and internal defects within the 3D heterostructure. This integrated characterization establishes a robust link between defect engineering and enhanced structural integrity, critical for optimizing the PEC properties of these semiconductor electrodes.

To investigate the impact of the synthesis process on electrode composition and structure, x-ray diffraction (XRD) analysis was performed on Si substrate, ZnBi, ZnBiP, and ZnBiPM electrodes, with results shown in Fig. 4A and B. Distinct phase markers (plum blossoms, spades, hearts, diamonds) denote different crystalline phases. For the ZnBi electrode, new peaks at 12.0°, 24.2°, 25.8°, 36.6°, 49.5°, 49.6°, 63.4°, and 77.9° align with the (001), (002), (011), (003), (004), (113), (005), and (006) planes of BiOCl (JCPDS No. 73-2060), while peaks at 11.6°, 36.7°, 63.3°, and 77.8° correspond to the (110), (420), (622), and (802) planes of Bi_2_O_3_ (JCPDS No. 74-1374), confirming successful growth of Bi_2_O_3_ and BiOCl on the Si/Zn substrate. In the ZnBiP electrode, additional peaks at 35.1° and 55.3° match the (111) and (200) planes of BP (JCPDS No. 73-1358), evidencing effective BP integration. In contrast, the ZnBiPM electrode shows no distinct Ti_3_C_2_T*_x_* peaks, likely due to its low Ti_3_C_2_T*_x_* content, rendering it below the detection threshold.

In Fig. 4B, an enlarged view of Fig. 4A reveals a rightward shift in BiOCl and Bi_2_O_3_ peaks for ZnBiP and ZnBiPM relative to ZnBi. This shift is attributed to high-temperature annealing during BP incorporation, which induces oxygen atom detachment from the BiOCl and Bi_2_O_3_ lattices, generating atomic-scale oxygen vacancies [12]. These vacancies contract the lattice parameters, shifting diffraction peaks to higher angles, consistent with defect-mediated structural evolution in these heterostructured electrodes. The XRD data underscore the precise control of phase composition and defect states of these materials in this work.

To elucidate the effects of BP nanosheets and Ti_3_C_2_T*_x_* on the surface structure of composite electrodes, Raman spectroscopy was employed to probe molecular and defect characteristics, with results presented in Fig. 4C to E. For the ZnBi electrode (Fig. 4C), prominent bands at 59.9 and 146 cm^−1^ are assigned to the external and internal Bi–Cl stretching modes of the A_1g_ symmetry, respectively, while a band at 202.4 cm^−1^ corresponds to the internal *E*_g_ stretching mode of BiOCl, consistent with prior reports [28–30]. Upon BP incorporation (ZnBiP; Fig. 4D), 2 additional peaks emerge at 362.3 and 392.5 cm^−1^, reflecting BP’s characteristic vibrational modes. The observable peaks of the ZnBiPM at 397.8 cm^−1^ correspond to the vibration of Ti–O–P. The appearance of the Ti–O–P peak also indicates the high stability of ZnBiPM [12], indicating the influence of MXene on the vibrational landscape. Additionally, a peak at 470.2 cm^−1^ in Fig. 4E is attributed to the Bi–O stretching mode of Bi_2_O_3_ [31], persisting across all samples. These Raman shifts reveal a progressive modification of the surface molecular structure, driven by the sequential integration of BP and MXene. The observed peak shifts and intensities suggest enhanced interlayer interactions and defect passivation within the heterostructure, aligning with the structural evolution observed in SEM and XRD analyses.

To probe the chemical bonding and surface interactions, particularly the anchoring of BP within the composite electrode, XPS was employed, with results shown in Fig. 4F to K and Fig. S2. All spectra were calibrated to the C 1s peak at 284.6 eV [32]. The survey spectrum (Fig. S2A) confirms the presence of Zn, Bi, O, Cl, Ti, C, and P in the ZnBiPM electrode. High-resolution Zn 2p and Cl 2p spectra of the ZnBi electrode (Fig. S2B and C) reveal Zn 2p_3/2_ and Zn 2p_1/2_ peaks at 1,022.6 and 1,045.6 eV, respectively, indicative of ZnO formation via air annealing, and Cl 2p_3/2_ and Cl 2p_1/2_ peaks at 197.8 and 199.4 eV, consistent with BiOCl. High-resolution Bi 4f and O 1s spectra of ZnBi (Fig. 4F and G) display Bi 4f_7/2_ peaks at 158.85 eV (BiOCl) and 159.35 eV (Bi_2_O_3_), and Bi 4f_5/2_ peaks at 164.15 eV (BiOCl) and 164.65 eV (Bi_2_O_3_) [31], while O 1s peaks at 529.85 and 532.1 eV correspond to Bi–O–Bi and adsorbed oxygen, respectively [33].

For the ZnBiPM electrode (Fig. 4H to K), the P 2p spectrum exhibits peaks at 132.55 and 133.3 eV (P 2p_3/2_ and P 2p_1/2_), with additional features at 134.35 eV (P–O) and 134.85 eV (P*_x_*O*_y_*), reflecting BP oxidation during spin-coating and annealing [11,12], and a peak at 133.85 eV (P–O–Ti) evidencing chemical bonding between BP and Ti_3_C_2_T*_x_* [12]. In the Bi 4f spectrum, a high-energy shift of BiOCl and Bi_2_O_3_ peaks, alongside new peaks at 158.7 and 164.1 eV (Bi–O–P) [34,35], confirms strong BP interactions with the Bi_2_O_3_/BiOCl substrate, facilitating defect repair at the interface. The O 1s spectrum reveals a new oxygen vacancy peak, attributed to lattice oxygen loss during high-temperature annealing. The Ti 2p spectrum displays peaks at 460.15 eV (Ti–C 2p_3/2_), 462.95 eV (Ti–O_x_ 2p_3/2_), 465.05 eV (Ti–O 2p_3/2_), 465.85 eV (Ti–C 2p_1/2_), 466.95 eV (Ti–O_x_ 2p_1/2_), and 468.9 eV (Ti–O 2p_1/2_), verifying Ti_3_C_2_T*_x_* incorporation and partial TiO_2_ formation [36]. These XPS data highlight the formation of robust interfacial bonds (e.g., P–O–Ti and Bi–O–P) and oxygen vacancies, which enhance structural stability and charge transfer within the heterostructure, aligning with the crystallographic and morphological evolution observed earlier. Meanwhile, the addition of BP and MXene causes the O 1s spectrum peaks (adsorbed O and Bi–O–Bi) and the Bi 4f spectrum peaks (Bi 4f_7/2_ and Bi 4f_5/2_) to shift toward higher binding energies. This transformation might be due to the interaction between BP and MXene as well as between Bi_2_O_3_ and BiOCl components, which reduces the electron density around Bi and O atoms.

**Fig. 5.**
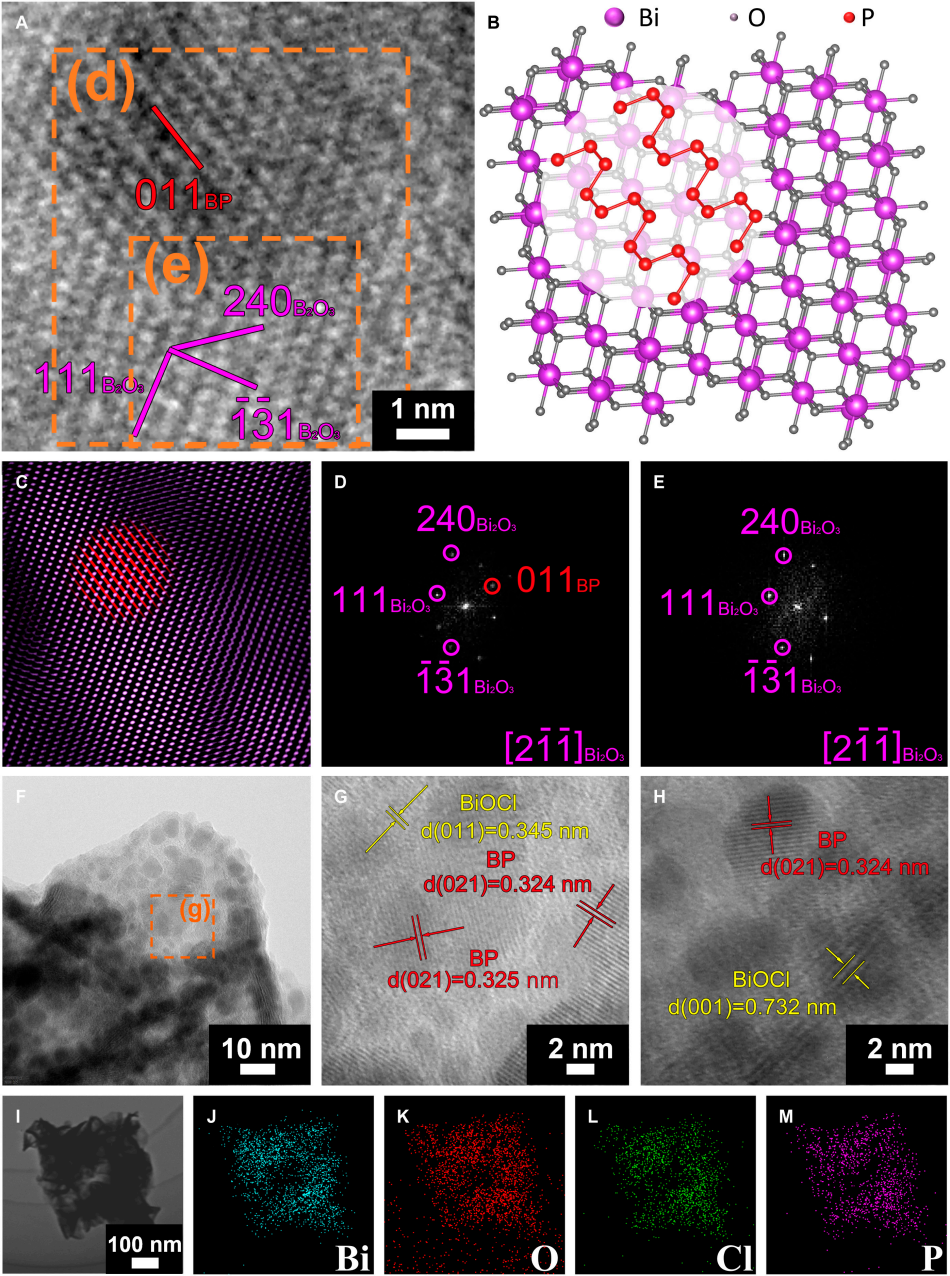
(A) The HRTEM image of ZnBiP sample and (B) the corresponding atomic model and (C) corresponding IFFT model (purple represents Bi_2_O_3_, red represents BP) and (D and E) the corresponding FFT patterns from the area marked by dashed square in (A). (F) HRTEM images of ZnBiP sample, with (G) of enlarged diagrams of (F). (H) HRTEM images of ZnBiP sample. (I to M) Morphology and EDX mapping images of P, Ti, C, and O elements derived from ZnBiP sample.

High-resolution transmission electron microscopy (HRTEM) was employed to examine the ZnBiP electrode, verifying the integration of BP nanostructures and their role in defect repair within the 3D hetero-framework, as depicted in Fig. 5A to H. The HRTEM image of ZnBiP (Fig. 5A) is complemented by an atomic structure schematic (Fig. 5B). The inverse fast Fourier transform (IFFT) image (Fig. 5C) highlights Bi_2_O_3_ (purple) and BP (red), with brightness intensity reflecting phase prevalence. BP predominates in regions of diminished Bi_2_O_3_ signal, suggesting that BP infiltrates and repairs Bi_2_O_3_ defects. Fourier transform (FFT) images of selected regions (Fig. 5D and E) identify BP with (011) facets and Bi_2_O_3_ with ($\bar{1}\bar{3}1$), (240), and (111) facets along the [2$\bar{1}\bar{1}$] zone axis, confirming BP’s anchoring within Bi_2_O_3_ defect sites. Further HRTEM analysis (Fig. 5F) and its magnified view (Fig. 5G) reveal lattice spacings of 0.345 nm, corresponding to BiOCl (011), and 0.324 nm, matching BP (021). These indicate BiOCl as the lighter substrate beneath dispersed BP flakes, forming a surface-dispersed heterostructure. Additional lattice spacings of 0.324 nm [BP, (021)] and 0.733 nm [BiOCl, (001)] in Fig. 5H reinforce BiOCl’s dominance in the underlying matrix. EDX from a selected region (Fig. 5I; spectra in Fig. 5J to M) detects Bi, O, Cl, and P, validating the compositional integrity of the ZnBiP heterostructure. These findings demonstrate BP’s dual role in filling internal defects and stabilizing surface interfaces, enhancing the structural coherence critical for PEC devices. It can also be observed that a large number of BP nanosheets are attached to the edges of the BiOCl layers, which may be the reason for the reduction in the pores of the porous structure (as shown in Fig. 5F).

Ultraviolet–visible (UV–Vis) spectroscopy was employed to evaluate the light absorption properties of the composite electrodes, with results presented in Fig. S3. The ZnBi electrode exhibits strong absorption in the low-wavelength UV region and the high-wavelength red region. The addition of BP and Ti_3_C_2_T*_x_* slightly enhanced the absorption capacity of the ZnBi electrode in the visible light region, as the layers of BP and Ti_3_C_2_T*_x_* were layered with ZnBi to repair the defects of the 3D heterogeneous framework, and BP and Ti_3_C_2_T*_x_* had well visible light absorption capacity.

### PEC performances

**Fig. 6.**
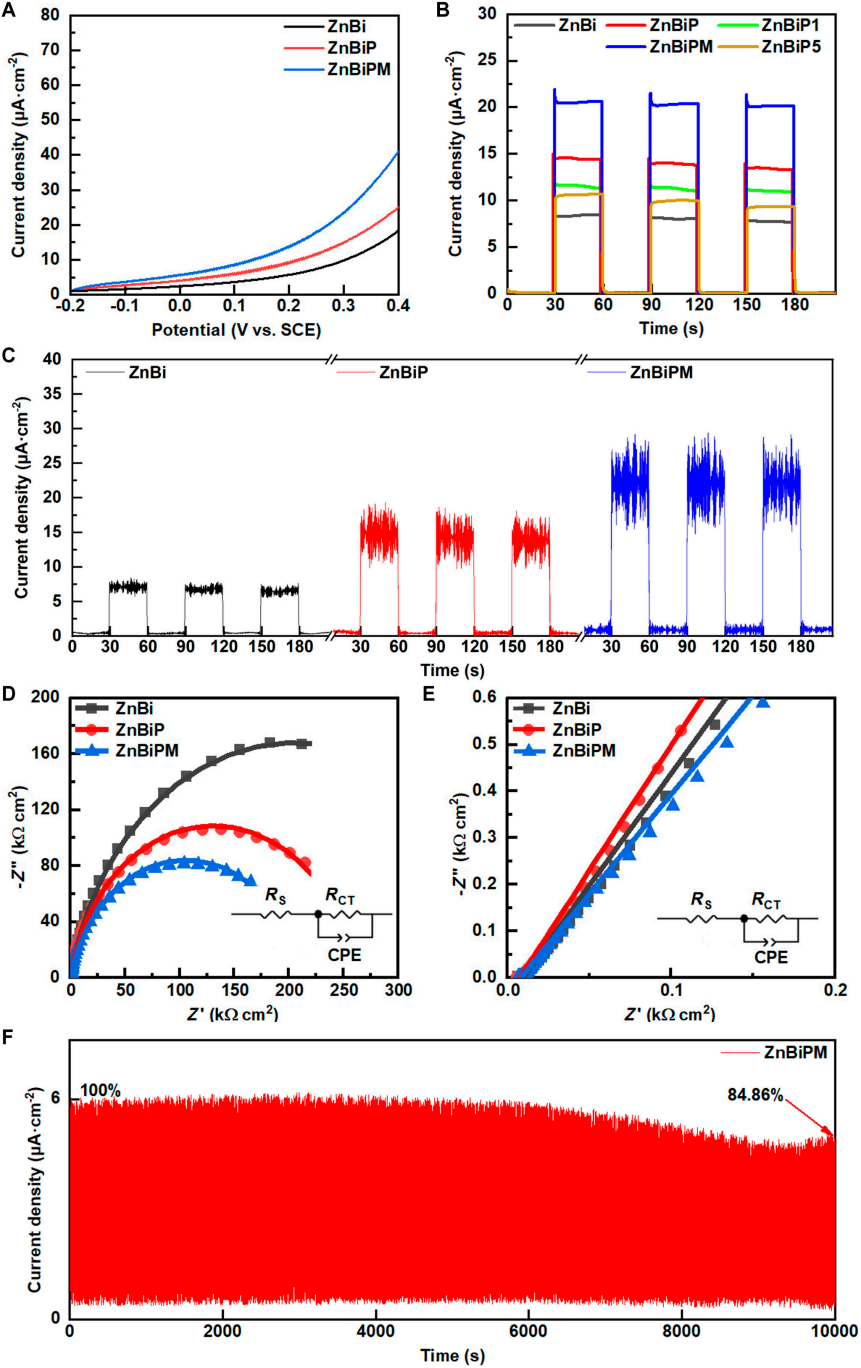
(A) LSV curves of ZnBi, ZnBiP, and ZnBiPM electrodes. (B) Transient current densities of ZnBi, ZnBiP, and ZnBiPM electrodes in light irradiation. (C) Transient current densities of ZnBi, ZnBiP, and ZnBiPM electrodes in AEF and light irradiation. (D) Nyquist plots of ZnBi, ZnBiP, and ZnBiPM electrodes (the lower right inset is the corresponding equivalent circuit model of ZnBi, ZnBiP, and ZnBiPM electrodes). (E) High-frequency partial enlarged detail of Nyquist plots for ZnBi, ZnBiP, and ZnBiPM electrodes (the lower right inset is the corresponding equivalent circuit model of ZnBi, ZnBiP, and ZnBiPM electrodes). (F) Device stability of ZnBiPM electrode in light irradiation.

To evaluate the PEC properties of the electrodes, we performed linear sweep voltammetry (LSV) measurements under illumination, as shown in Fig. 6A. Photocurrent density rises with increasing bias voltage across all electrodes. The ZnBiP electrode exhibits a higher photocurrent response than the ZnBi electrode over the −0.2- to 0.4-V range [versus saturated calomel reference electrode (SCE)], with further enhancement observed in the ZnBiPM electrode upon addition of a Ti_3_C_2_T*_x_* layer. This enhancement phenomenon is due to the presence of porous defects within the 3D hetero-framework, which hinders the transport of charge carriers, but the hierarchical integration of BP and Ti_3_C_2_T*_x_* nanosheets effectively fills these porous defects, expanding the interfacial contact area and increasing the density of active sites. Furthermore, the annealing process generates oxygen vacancies, which introduce defect levels below the conduction band (CB) minimum. These defect levels narrow the band gap and act as electron traps, facilitating the migration of photogenerated electrons. Simultaneously, the formation of oxygen vacancies at the interface enhances the built-in electric field strength. This intensified field drives the directional separation of electrons and holes, consequently increasing the photocurrent.

To assess the PEC performance of ZnBi, ZnBiP, ZnBiPM, and ZnBiP1/ZnBiP5 electrodes (coated with BP once or 5 times via spin-coating), we evaluated transient photocurrent responses under a 60-s switching cycle with illumination, as shown in Fig. 6B. The ZnBiP electrode exhibits an average transient current density of 14.55 μA cm^−2^, an increase of 1.7 times over ZnBi (8.38 μA cm^−2^). This enhancement arises from BP nanosheets filling porous defects within the 3D hetero-framework, augmenting active sites alongside oxygen vacancies induced by annealing. Additionally, Bi–O–P bonds form an electron transport channel, facilitating efficient separation and transfer of photogenerated carriers. In contrast, ZnBiP1 and ZnBiP5 electrodes display lower current densities, underscoring an optimal BP loading in ZnBiP, where excessive or insufficient BP compromises performance. The ZnBiPM electrode achieves a current density of 20.46 μA cm^−2^, 1.4 times higher than ZnBiP, attributed to Ti_3_C_2_T*_x_* further repairing defects and enhancing conductivity. As shown in Table S2 [24,37–39], compared with other literatures, this electrode has achieved higher PEC performance in transient photocurrent, which may be attributed to the construction of 3D and 2D composite nanostructures, defect regulation, and the formation of heterojunctions. The metallic character of Ti_3_C_2_T*_x_* acts as a co-catalyst, significantly boosting charge transport in the hybrid structure. These findings highlight the synergistic roles of defect engineering and conductive layer integration in optimizing PEC efficiency, with ZnBiPM emerging as a superior photoelectrode design.

**Fig. 7.**
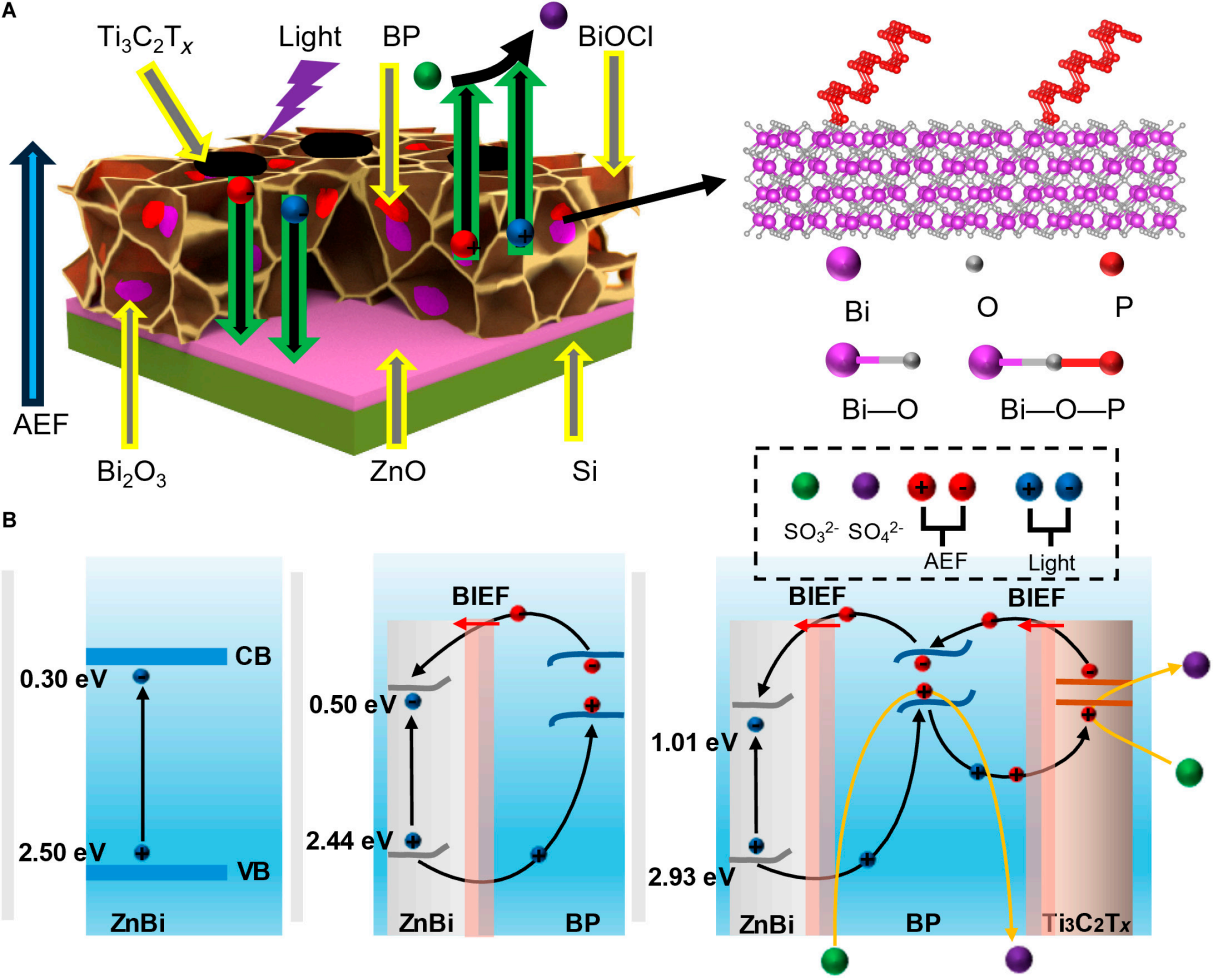
(A) Structure diagram of the device. (B) Charge transfer path schematic diagram of ZnBi, ZnBiP, and ZnBiPM.

The AEF response characteristics of ZnBi, ZnBiP, and ZnBiPM electrodes were evaluated under combined AEF and illumination, as depicted in Fig. 6C. Compared with ZnBi, the amplitude increased by approximately 102.7% and 221.2%, respectively, after adding BP and MXene. Under dual stimulation, the response amplitude exceeded the individual AEF response amplitude or the light field response amplitude, indicating that there is a synergistic enhancement effect between AEF and light. At the same time, we observed that the amplitude of current density fluctuated around the baseline. This is because AEF is applied to the electrodes through the wireless charger. Therefore, the direction of AEF is not constant but changes in a cyclical manner. The current currently generated also fluctuates periodically to a certain extent. When an electric field is applied along the direction from Zn to MXene (the AEF direction), electrons flow opposite to the field, establishing a fixed charge distribution within the material. Consequently, near the contact interface, a built-in electric field forms, oriented opposite to the applied AEF. This built-in field increases the potential energy in regions of electron accumulation, causing upward band bending. Conversely, it decreases the potential energy in regions of hole accumulation, resulting in downward band bending. This configuration produces the energy band structure depicted in Fig. 7, thereby enhancing the generation and separation of electron–hole pairs. When BP and Ti_3_C_2_T*_x_* are incorporated, they hierarchically fill the porous defects in the 3D hetero-framework. Their high conductivity amplifies the induction of AEF, thereby enhancing charge dynamics. Compared with the AEF of 1,100 V m^−1^ for WO_3_/Bi_10_O_6_S_9_/BP and WO_3_/BiVO_4_/MXene, with coupling amplification factors (the ratio of coupled current density of AEF and light illumination to the current density under illumination) of 1.6 and 1.2, respectively, in this work, the coupling amplification factor is 1.4. A similar or even stronger coupling amplification factor can be achieved with a lower AEF. For specific data, please refer to Table S3 [10,11]. Under the combined action of 1,100 V m^−1^ AEF and light, the photocurrent of the WO_3_/BiVO_4_/MXene (Ti_3_C_2_T*_x_*) heterojunction can reach 1.39 mA cm^−2^. However, in this experiment, under the action of only 30-W blue light, the photocurrent can reach 20.46 μA cm^−2^. Meanwhile, by using smaller AEF and light field coupling, the coupling current reached 29 μA cm^−2^, and similar coupling amplification capabilities can be achieved. This might be attributed to the defect regulation that repaired the surface hole defects. Through this repair process, 2D materials constantly repair defects and construct dispersed heterojunction structures, enhancing the coupling effect of the sensor. These results underscore the critical role of defect repair and conductive additives in maximizing PEC performance under external field modulation.

In order to analyze the charge transfer dynamics of the electrochemical system, electrochemical impedance spectroscopy (EIS) was conducted on the electrodes, with results presented in Fig. 6D and E. The equivalent circuit, modeled using ZView software (inset), includes *R*_S_ (internal resistances of the series electrodes), *R*_CT_ (charge transfer resistance at interfaces) [11,40], and CPE (constant phase element at the electrode–electrolyte interface) [12,41]. The ZnBiPM electrode exhibits the lowest *R*_CT_ (211 kΩ cm^2^) and negligible *R*_S_, signifying the fastest interfacial charge transfer among the tested electrodes. The incorporation of BP and Ti_3_C_2_T*_x_* repairs porous defects in the 3D hetero-framework and introduces Bi–O–P bonds, establishing an efficient carrier transport channel. This structural optimization markedly reduces *R*_CT_, enhancing charge mobility and underscoring the efficacy of defect engineering in improving the device performance.

The stability of the ZnBiPM electrode under light irradiation was assessed over 1,000 cycles (10,000 s total, 10 s switching cycle), as shown in Fig. 6F. The electrode retains 84.86% of its initial photocurrent. Meanwhile, the stability of the ZnBiPM electrode under light irradiation was tested for 24 h, and the final stability performance reached 57.27%, demonstrating a relatively certain degree of stability, as shown in Fig. S4 [10,12,42]. The decrease in stability might be due to the oxidation of BP, which leads to the loss of active substances. At the same time, during the charging and discharging process of the test, the Ti^3+^ ions are oxidized to TiO_2_, causing the MXene layers to restack, reducing the transmission channels, and the detachment of the surface BP from MXene. As shown in Table S3 [10,11], compared with other literatures, this work achieves higher stability over a longer period of time. This might be due to the defect regulation of the 3D and 2D composite nanostructure, which stabilizes the formed chemical bonds and stabilizes the device performance. The Ti_3_C_2_T*_x_* on the outer layer reduces the oxidation of BP. These findings highlight the efficacy of hierarchical defect repair and protective coating strategies in enhancing long-term device reliability.

### Working mechanism

The operational mechanism of the ZnBiPM electrode was elucidated through its energy level structure, depicted in Fig. 7A. The schematic illustrates BP and Bi_2_O_3_ interconnected via Bi–O–P bonds, with defects precisely repaired within the heterostructure. Experimental potentials were measured against the SCE, while band positions are referenced to the normal hydrogen electrode (NHE), with NHE being 0.24 eV above SCE [43].

The bandgap was obtained from the Tauc plots converted by the UV–Vis diffuse reflection spectrum (Fig. S5A). Then, the UV photoelectric spectra of ZnBi, ZnBiP, and ZnBiPM were measured under the excitation of 21.21 eV to obtain the valence-band (VB) maximums (Fig. S5B). By using the linear approximation method, the VB maximum of ZnBi, ZnBiP, and ZnBiPM was 2.50, 2.44, and 2.93 eV, respectively (versus NHE, pH 0).

Under 405-nm laser irradiation, BiOCl generates abundant photogenerated electron–hole pairs. Electrons transition to the CB, while holes remain in the VB. Due to direct contact between BP, BiOCl, and Ti_3_C_2_T*_x_*, and the lower VB of BP (1.17 eV) relative to BiOCl (2.47 eV), holes transfer efficiently from BiOCl’s VB to BP’s VB and subsequently to Ti_3_C_2_T*_x_*. Concurrently, where BP interfaces with Bi_2_O_3_ and BiOCl, the VB alignment (BiOCl: 2.47 eV > Bi_2_O_3_: 1.98 eV > BP: 1.17 eV) drives hole migration from BiOCl to Bi_2_O_3_, then to BP, ultimately reaching the electrolyte interface. Here, SO₃^2−^ acts as a hole scavenger, oxidizing to SO₄^2−^ and mitigating electron–hole recombination. Photogenerated electrons in BiOCl’s CB (−0.51 eV) transfer to Bi_2_O_3_’s CB (−0.44 eV) due to favorable band alignment at their interface. Subsequently, electrons in Bi_2_O_3_’s CB migrate to ZnO’s CB (−0.31 eV) in regions of contact, reaching the electrode interface. Based on these results, the electron transfer path of ZnBiPM is shown in Fig. 7B. The energy band diagram (Fig. S5C) details the CB and VB positions: BP at −0.83 eV (CB) and 1.17 eV (VB) with a 2.00-eV bandgap [44], BiOCl at −0.51 eV (CB) and 2.47 eV (VB) with a 2.98-eV bandgap [23], Bi_2_O_3_ at −0.44 eV (CB) and 1.98 eV (VB) with a 2.42-eV bandgap [45], ZnO at −0.31 eV (CB) and 2.89 eV (VB) with a 3.2-eV bandgap [46], and the Fermi level of Ti_3_C_2_T*_x_* at −0.17 eV (versus NHE) [12]. This cascade, facilitated by the heterostructure’s defect-repaired architecture and Bi–O–P bonding, enhances charge separation and transfer efficiency, underpinning the ZnBiPM electrode’s superior PEC performance.

Based on the previous research, the introduction of BP into BiOCl leads to the formation of oxygen vacancies. Therefore, we obtained the band structure and density of state diagrams of the intrinsic BiOCl and BiOCl with one oxygen vacancy through first-principles calculations. The BiOCl obtained from the calculations is an indirect bandgap semiconductor with a bandgap value of 2.58 eV, as shown in Fig. S6A. At the same time, according to the state density analysis (Fig. S6B), the VB of BiOCl is mainly contributed by the 3p orbitals of Cl and the 2p orbitals of O, while the CB is mainly contributed by the 6p orbitals of Bi. When an oxygen vacancy is introduced into the system, an impurity energy level appears between the VB and the CB, thereby reducing the bandgap, which is reduced to 1.16 eV as shown in Fig. S6D. Through the analysis of the state density diagram (Fig. S6E), it can be known that when oxygen vacancies exist, the impurity energy level introduced is mainly contributed by the 6p, 3p, and 2p orbitals of Bi, Cl, and O, respectively. As shown in Fig. 7B, after adding BP to ZnBi, the bandgap decreases from 2.20 eV to 1.94 eV, which is consistent with the above theoretical calculation results. The numerical differences were due to the energy bands of the substrate material and the composite structure in the experiment, which were different from those in the theoretical model.

The influence of AEF direction on charge carrier dynamics was further explored within the ZnBiPM electrode. When the AEF is oriented from ZnO to MXene, electron–hole pairs induced by AEF are generated in the BP and Ti_3_C_2_T*_x_* layers. Electrons migrate sequentially through Ti_3_C_2_T*_x_*, BP, BiOCl, and Bi_2_O_3_, ultimately reaching ZnO, while holes move toward BP and Ti_3_C_2_T*_x_*, flowing into the electrolyte. This AEF-driven charge migration aligns with the direction of light-induced carriers, synergistically combining with the built-in electric field from applied bias to enhance photogenerated carrier transport.

Conversely, when the AEF is directed from Ti_3_C_2_T*_x_* to ZnO, AEF-induced electron–hole pairs in BP and Ti_3_C_2_T*_x_* follow an opposite path: Holes traverse Ti_3_C_2_T*_x_*, BP, BiOCl, and Bi_2_O_3_ toward ZnO, while electrons flow to BP and Ti_3_C_2_T*_x_*, reaching the electrolyte. This opposes the light-induced carrier direction, counteracting the built-in electric field and impeding photogenerated carrier migration. Consequently, photocurrent enhancement or reduction depends on AEF orientation, with the ZnO-to-Ti_3_C_2_T*_x_* direction amplifying performance and the reverse weakening it. This interplay highlights a synergistic light–AEF effect, where illumination further boosts the AEF response, optimizing charge separation and transfer in the heterostructure.

The built-in electric field amplifies the AEF effect, enhancing signal strength in the ZnBiPM electrode. High-temperature annealing induces oxygen atom detachment from the lattice, generating abundant oxygen vacancies at the atomic scale. These vacancies serve dual roles: accelerating carrier migration and acting as active sites for electron capture, thereby boosting PEC efficiency. The BP further enhances performance through multiple mechanisms. It forms a heterojunction with Bi_2_O_3_, expanding carrier transport pathways and suppressing electron–hole recombination. Concurrently, BP anchors to Bi_2_O_3_ via Bi–O–P bonds, repairing defects in the 3D hetero-framework, increasing the electrode’s effective contact area, and modulating active site density. Additionally, BP’s high conductivity enhances AEF-induced carrier separation, synergistically optimizing charge dynamics and underscoring its pivotal role in the heterostructure’s superior performance.

To explore the practical utility, a sEMG sensor was developed to monitor human sEMG signals under light stimulation. Testing was performed in a consistent enclosed environment, where the sensor, mounted on the human arm, exhibited stable and regular sEMG signals, suggesting minimal environmental interference. Figure S7A to D illustrates the relaxed and clenched states of the arm under 2 conditions: placed flat on the table and suspended on one side. When stimulated by light, the average amplitude of the signal increased by approximately 30% to 40%. Concurrently, fluctuations in the signal were observed during the tests. It is speculated that these fluctuations might originate from muscle contraction, demonstrating the sensitivity of the sensor within a certain range. Furthermore, the results have demonstrated the potential application scenarios of the light-induced enhancement effect on the sEMG signal. Furthermore, the sensitivity, resolution, and range of the sensors were also tested. The detailed results can be found in Table S5. The schematic diagram of PEC and AEF performance tests and the internal structure of the wireless charger are shown in Fig. S8. This mutual reinforcement of sEMG and PEC effects highlights the potential of leveraging amplified voltage signals for precise sEMG measurement, demonstrating the ZnBiPM electrode’s efficacy in bioelectronic applications.

## Conclusion

This work introduces the ZnBiPM electrode, a defect-engineered photoelectrode fabricated by sequentially depositing nanomaterial layers onto a Si substrate via electrochemical displacement, spin-coating, vacuum annealing, and ozone treatment. Under a bias of 0.3 V (versus SCE) in 0.5 M Na₂SO_3_, the ZnBiPM electrode exhibits an average transient photocurrent density of 20.46 μA cm^−2^, which is 1.4 times higher than that of the ZnBiP electrode. The successive integration of BP and Ti_3_C_2_T*_x_* disperses these materials across the BiOCl surface, forming heterostructures, while infiltrating larger pores to hierarchically fill and repair nanoscale defects within the underlying BiOCl/Bi_2_O_3_ 3D hetero-framework. This enhances electrode contact area and active site density, markedly improving PEC performance and AEF synergy. The combined AEF–light stimulation elicits a stronger electrical response than AEF alone, corroborated by a significant sEMG signal enhancement under illumination in practical testing. Moreover, the electrode retains 84.86% stability after 10,000 s of cyclic operation, reflecting robust structural integrity attributed to defect repair and Bi–O–P bonding. These findings establish a rigorous framework for defect engineering in heterostructured photoelectrodes, demonstrating how hierarchical defect repair and conductive layer integration can synergistically enhance PEC and electrostatic field responses. This work offers a suggestion for designing high-performance, stable photoelectrodes, with broad implications for bioelectronic sensing and optoelectronic applications.

## Materials and Methods

### Materials and reagents

Dimethyl sulfoxide (DMSO) was procured from Sinopharm Chemical Reagent Co. Ltd., and BiCl_3_ was procured from Shanghai Macklin Biochemical Co. Ltd. The 2D BP nanosheet dispersion (0.1 to 5 μm lateral size, 1 to 10 layers, 0.2 mg ml^−1^) was obtained from Jiangsu XFNANO Materials Tech Co. Ltd. Multi-layer MXene powder was sourced from Beijing Beike New Materials Technology Co. Ltd. N-doped Si wafers (<100> orientation, 500 μm thickness, resistivity < 0.005 Ω cm, single-side polished) were purchased from Jining Jingxi Electronic Co. Ltd. Deionized water (resistivity > 18.2 MΩ cm) was produced using a Millipore purification system and used throughout.

### Preparation of the ZnBiPM electrode

Si wafers were sectioned into 1 × 2 cm^2^ pieces, sequentially ultrasonicated in deionized water, acetone, and ethanol (10 min each), dried under N_2_ flow, and plasma-etched (60 W, 5 min). The Zn films were deposited onto the Si substrates via magnetron sputtering to form Zn electrodes.

A 0.03 mol BiCl_3_ powder was dissolved in 100 ml of DMSO, and the Zn electrode was immersed at ambient temperature for 20 min. Galvanic replacement (2Bi^3+^ + 3Zn → 2Bi + 3Zn^2+^) facilitated Zn dissolution and Bi nucleation on the Si surface. The Bi-coated electrode was then soaked in ethanol (gas chromatography grade, ≥99.7%, H_2_O ≤ 0.2%) for 30 min to remove residual DMSO, triggering hydrolysis (BiCl_3_ + H_2_O → BiOCl + 2HCl) and forming a ZnBi electrode. The ZnBi electrode was dried at 60 °C under vacuum for 3 h, annealed at 250 °C in air for 30 min, and UV-irradiated for 10 min.

The BP nanosheets (0.25 mg ml^−1^) were spin-coated onto the ZnBi electrode (3,000 rpm, 30 s), and this process was repeated 3 times to form the ZnBiP electrode, followed by annealing at 300 °C in a vacuum glovebox for 30 min. Separately, 10 mg of MXene powder was dispersed in 50 ml of deionized water, ultrasonicated (1 h, 5 s on/5 s off) using a probe sonicator, and spin-coated (3,000 rpm, 30 s) onto the ZnBiP electrode to form the ZnBiPM electrode. The final electrode was annealed at 300 °C in a vacuum glovebox for 30 min.

### Material characterization

Morphologies and microstructures were examined via SEM (JEOL-7800F). EDX and HRTEM were performed on a JEOL JEM-2100Plus (200 kV, ambient temperature). Crystal structures were analyzed using a PANalytical Empyrean x-ray diffractometer. HRTEM images were processed with RADIUS software via fast FFT filtering. Raman spectra were collected on an inVia-Reflex confocal spectrometer (532 nm laser). Surface chemical states were assessed by XPS (ESCALAB 250Xi). Contact angles were measured with a Kono SL200B meter, and AFM images were acquired on an AFM5500M.

For AEF and light experiments, a 30-W, 405-nm blue laser (Guangzhou Yapusi Optoelectronics Technology Co. Ltd.) and a 30-W wireless charger (AEF source) were employed. The PEC properties, including the LSV, current versus time plot (*I*–*t*), EIS, and *I*–*t* stability, were evaluated in 0.5 M Na_2_SO_3_ electrolyte using a CHI 760E electrochemical workstation (Chenhua, Shanghai) under 30-W laser illumination and a 30-W AEF source. Measurements utilized a 3-electrode system (Pt counter electrode, SCE, and working electrode of the as-prepared samples) on Si substrates. *I*–*t* curves were recorded at 0.3-V bias, LSV was recorded from −0.2 to 0.4 V at varied scan rates, and EIS was recorded from 0.01 Hz to 100 kHz (5-mV amplitude). The setup was housed in a sealed iron box to eliminate external electrostatic and light interference.

## Data Availability

The datasets supporting the conclusions of this article are included within the article and its supplementary materials. Additional data, including raw sequencing data and experimental results, are available from the corresponding authors upon reasonable request.
