# Supplementary material for: Hierarchically Converged Defect Engineering with 2-Dimensional Black Phosphorus/MXene Sequence for Sensitive Photoelectrochemical-Electrostatic Sensors
Source: Research (Wash D C). 2025 Oct 24;8:0966. doi: 10.34133/research.0966 (PMC12550280; doi:10.34133/research.0966)
Supplement: Supplementary 1 — Figs. S1 to S8 Tables S1 to S5 [file research.0966.f1.docx]

Supporting information

**Hierarchically-converged defect engineering with 2D black phosphorus/MXene sequence for sensitive photoelectrochemical-electrostatic sensors**

*Wei Zeng ^a^, Yuan Zhang ^a^, Zhengyin Wu ^a^, Yumin Wang ^c^, Zichu Zhang ^a^, Liting Deng ^a^, Yuan Tian ^a^, Mengying Che ^a^, Yiming Chen ^a^, Pengfei Fang ^c^, Yi Xiong ^b,^*,* *Yun Tang ^b,^*, Shuoxue Jin ^d,^** *, Suiting Ning ^e^*

^a^ National Key Laboratory of Opto-Electronic Information Acquisition and Protection Technology, Anhui University, Hefei, 230601, Anhui, China

^b^ Analytical and Testing Center, School of Bioengineering and Health, School of Electronics and Electrical Engineering, Hubei Provincial Engineering Research Center for Wide-Bandgap Semiconductor Materials and Devices, Wuhan Textile University, Wuhan, 430073, Hubei, China

^c^ School of Physics and Technology, Key Laboratory of Nuclear Solid State Physics Hubei Province, Wuhan University, Wuhan 430072, China

^d^ Multi-disciplinary Research Division, Institute of High Energy Physics, Chinese Academy of Sciences, 100049, Beijing, China

^e^ School of Science, Hubei University of Technology, 430068, Wuhan, China

**Corresponding authors**

*E-mail: xiong@wtu.edu.cn (Yi Xiong)

*E-mail: ytang@wtu.edu.cn (Yun Tang)

*E-mail: jinshuoxue@ihep.ac.cn (Shuoxue Jin)


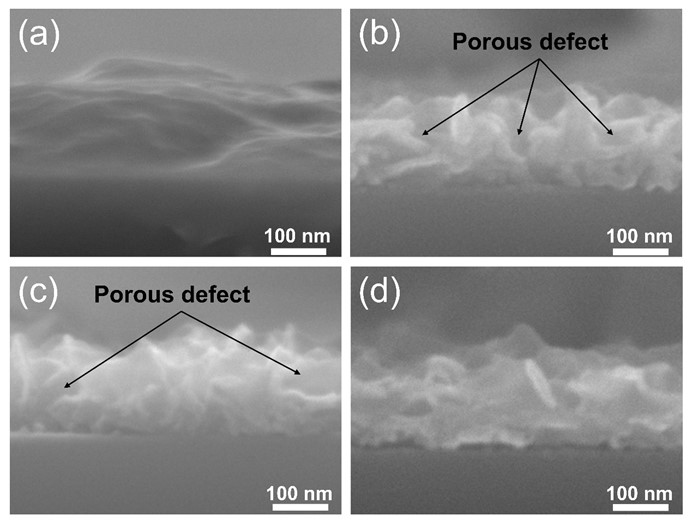


**Figure S1** (a-d) The cross-sectional SEM images of Si/Zn, ZnBi, ZnBiP, ZnBiPM.


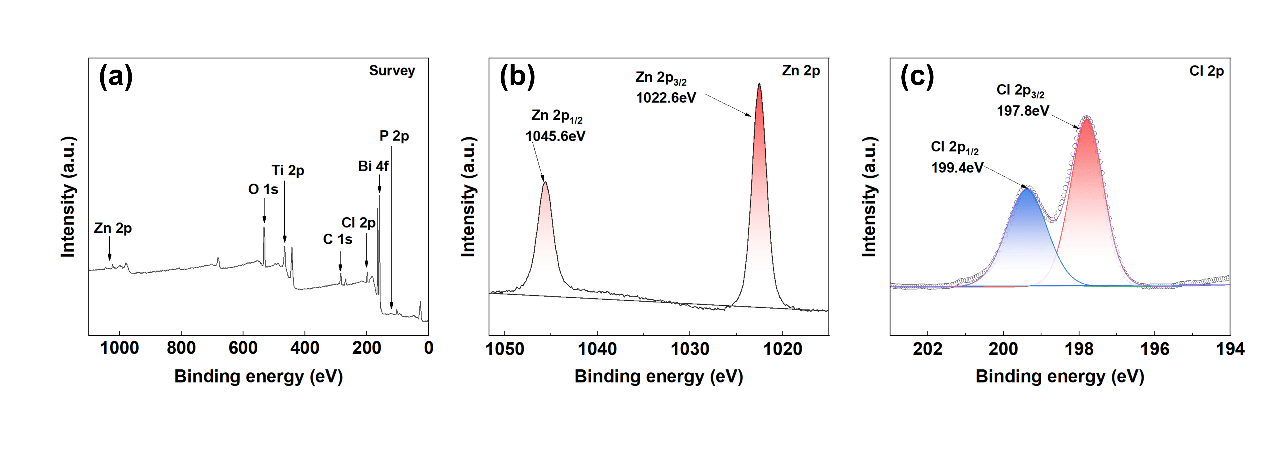


**Figure S2** (a) XPS survey scan of the ZnBiPM electrode. (b-c) XPS spectra of the ZnBi electrode: high-resolution XPS spectra of (b) Zn and (c) Cl.


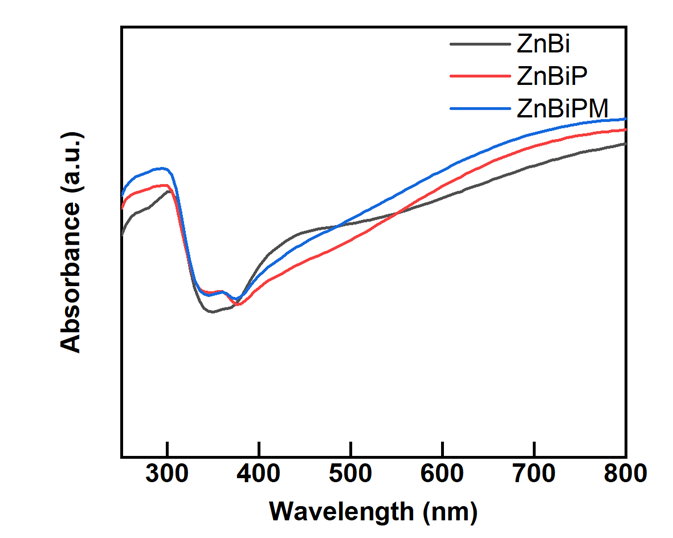


**Figure S3** UV-Vis spectra of the ZnBi, ZnBiP and ZnBiPM electrodes.


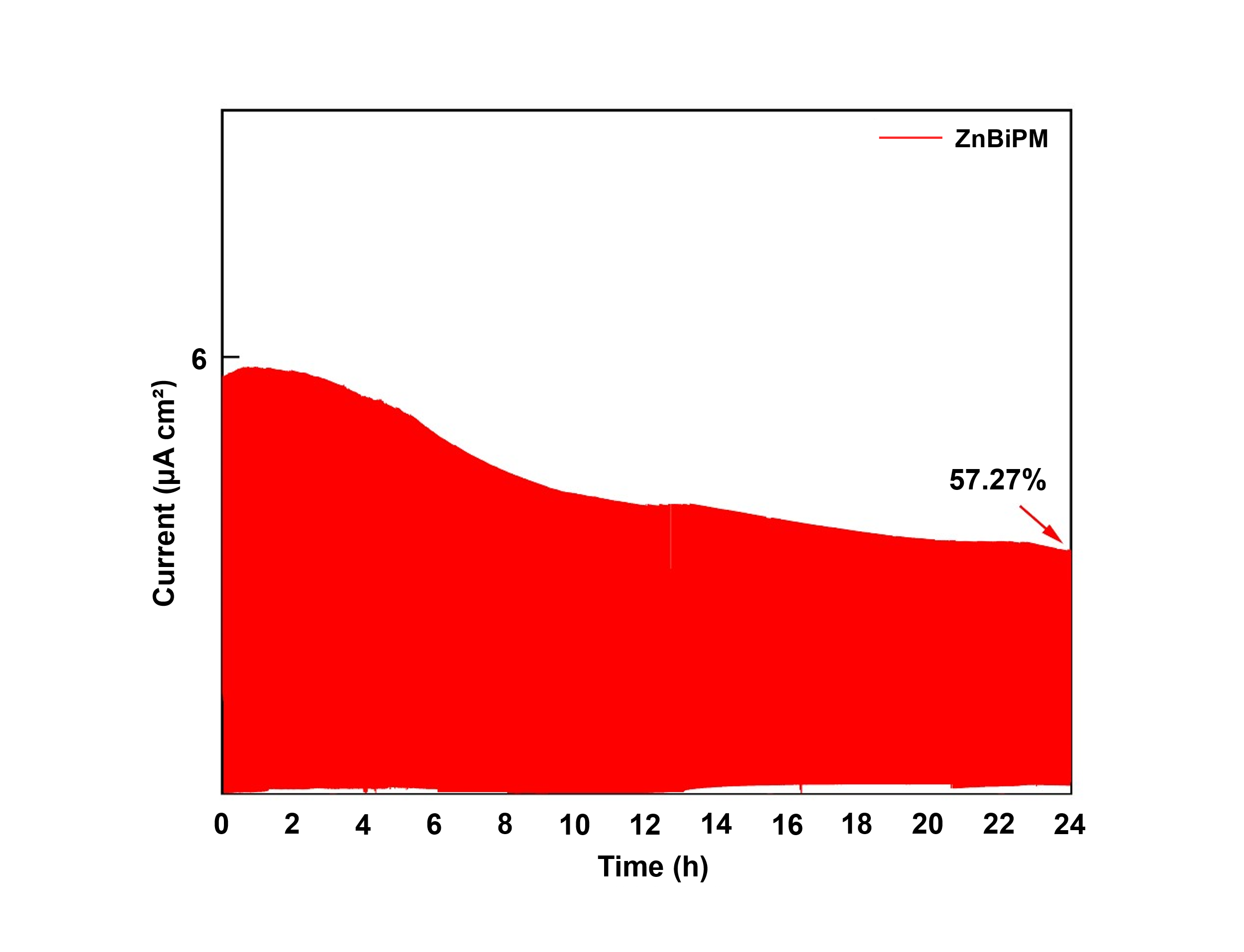
**Figure S4** Device stability of ZnBiPM electrode under 24-hour light irradiation.


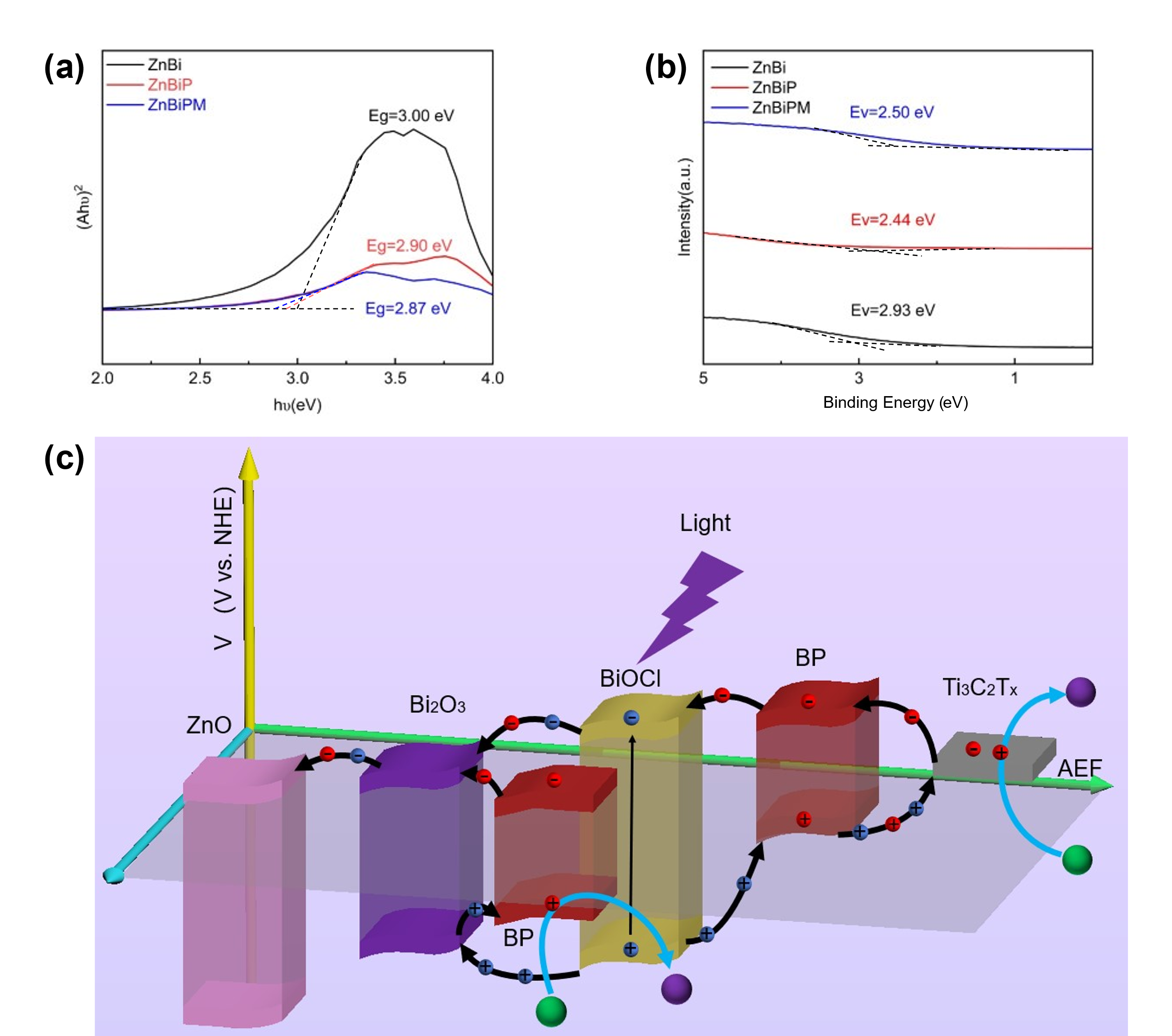


**Figure S5** (a) Tauc polts for ZnBi, ZnBiP and ZnBiPM using ${(F\left( R \right)h\nu)}^{1/2}$ as a function of the photon energy. (b) UV photoelectron spectra of ZnBi, ZnBiP and ZnBiPM. (c) Schematic diagram for analyzing charge transport paths of ZnBiPM electrode.


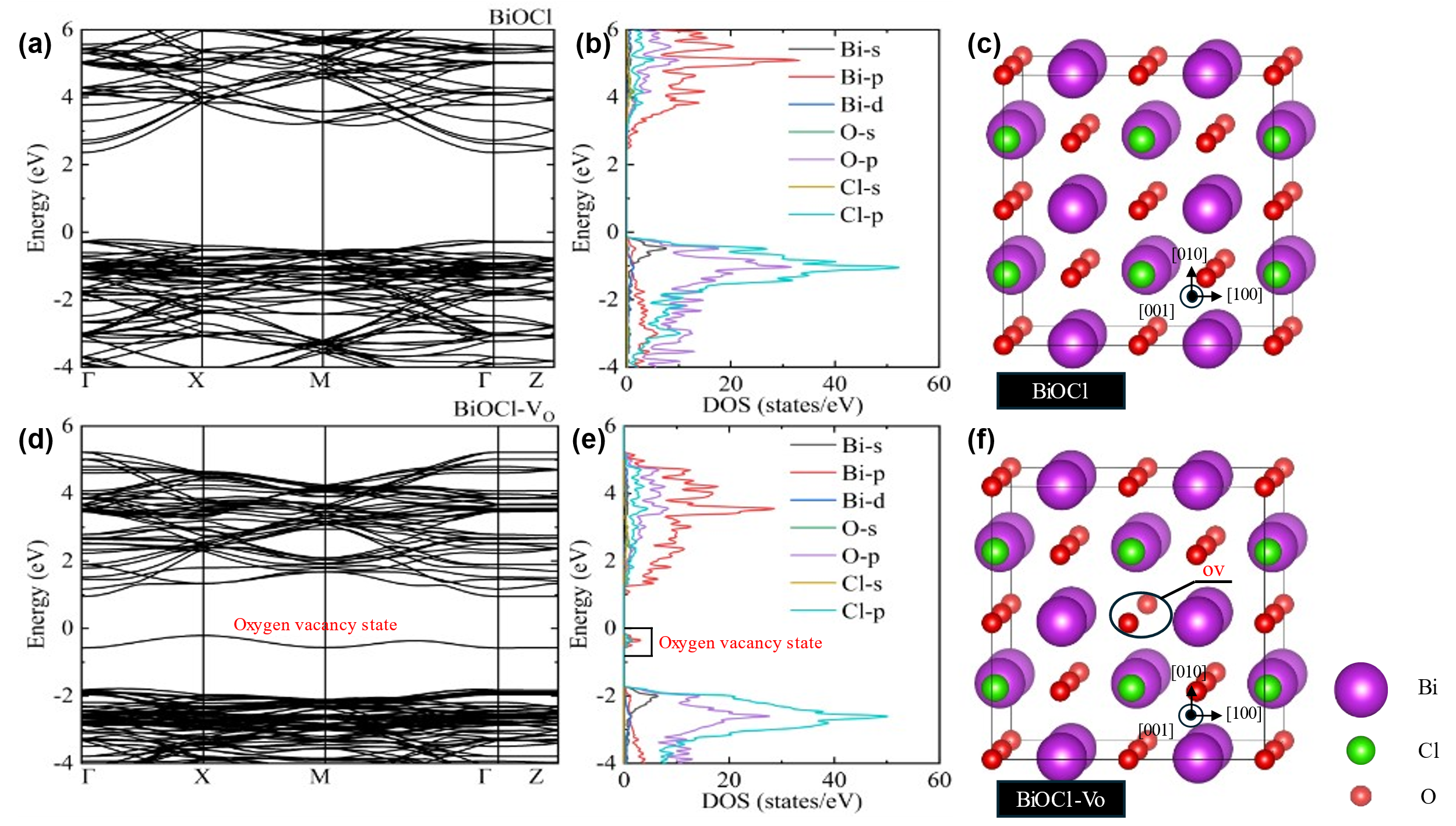


**Figure S6** (a) Band structure and (b) projected density of states (PDOS) for pristine BiOCl and (c) crystal structures of pristine BiOCl sample. (d) Band structure and (e) PDOS for BiOCl-V_O_ and (f) crystal structures of BiOCl-V_O_ sample.

The electronic band structure of BiOCl is performed by a first principles method, as implemented in the Vienna ab initio simulation package (VASP) based on density functional theory. The generalized gradient approximation (GGA) is used to deal with the exchange correlation energy. A quasi-Newton algorithm is used to deal with the relaxation and the force convergence criteria of structure relaxation is 0.001 eV Å^-1^. Lattice constants and internal atomic positions are fully relaxed. The cut-off energy for the plane wave basis is set to 500 eV. To evaluate the influence of oxygen vacancies on the band structure, we used a 2×2×2 supercell for the calculation. The corresponding k-point grid is set to 6×6×3 using the original Monkhorst–Pack scheme in the first Brillouin zone.

To determine the stability of the system, we calculated the corresponding elastic constants, and the results are shown in the Table S1. For BiOCl, its structure belongs to the tetragonal crystal system and there are six independent elastic constants. When the elastic constants satisfy the following stability criterion (C_11_ > 0, C_33_ > 0, C_44_ > 0, C_66_ > 0, (C_11_ - C_12_) > 0, (C_11_ + C_33_ - 2C_13_) > 0, [2(C_11_ + C_12_) + 2C_33_ + 4C_13_)] > 0), the system can be determined to be mechanically stable [1,2]. The calculated elastic constants of both pristine BiOCl and BiOCl with an oxygen vacancy satisfy the stability criteria, demonstrating the mechanical stability of both systems.

**Table S1 The elastic constants**

| C_ij_ | C_11_ | C_12_ | C_13_ | C_33_ | C_44_ | C_66_ |
| --- | --- | --- | --- | --- | --- | --- |
| BiOCl | 120.61 | 57.00 | 18.02 | 16.66 | 15.34 | 52.30 |
| BiOCl-V_O_ | 106.25 | 55.05 | 13.86 | 11.23 | 12.06 | 46.21 |


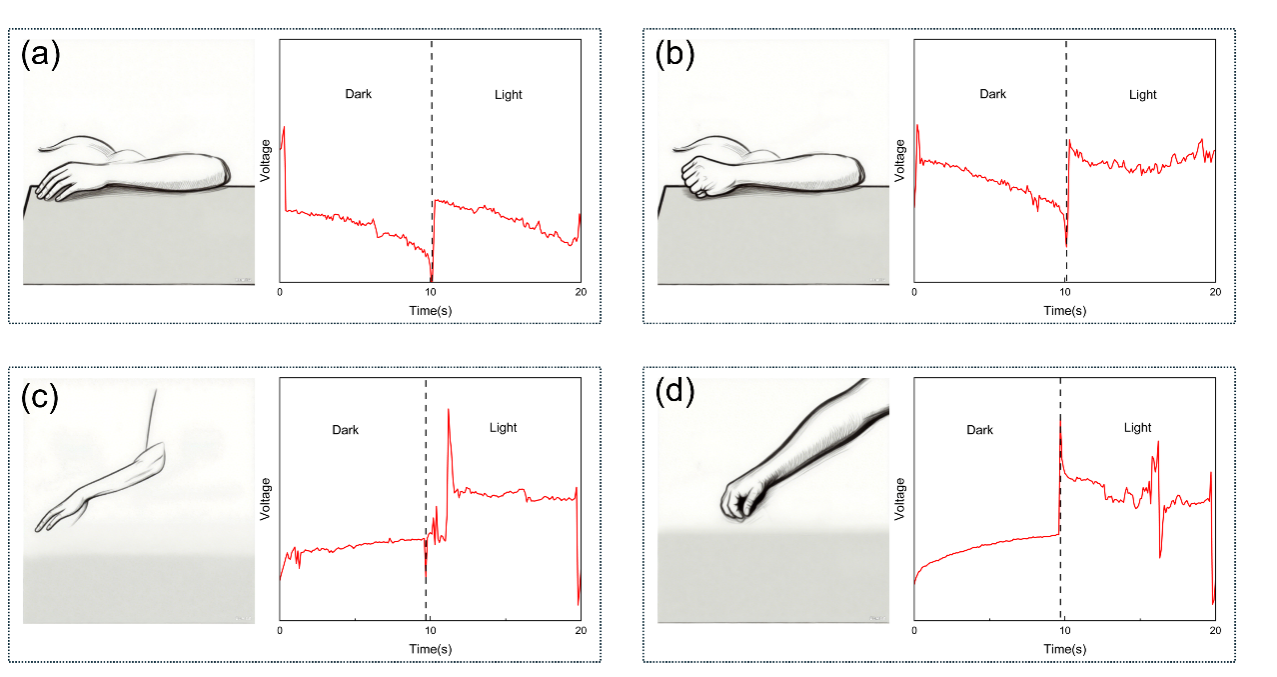


**Figure S7** (a) The arm is relaxed on the table, (b) the arm is clenched on the table, (c) the arm is relaxed hanging at the side of the body, (d) the arm is clenched hanging at the side of the body.

The schematic diagram of the test is shown in Figure S8a. During the test, we used a wireless charger with a power of 30 W as the AEF source, which is a contactless device. When the wireless charger is powered on, alternating current flows through the coil inside the charging plate. According to Ampere's Law and Faraday's Law of electromagnetic induction, a changing magnetic field is generated around the coil with current flowing through it. The alternating magnetic field generated by the transmitting end will pass through the sensor. According to Faraday's Law of electromagnetic induction, the changing magnetic field will generate an induced electromotive force in the sensor. Since the voltage input to the wireless charger is 220V, 50Hz alternating current, the direction of the electric field generated inside the sensor also changes periodically. The internal structure of the wireless charger is shown in Figure S8b.


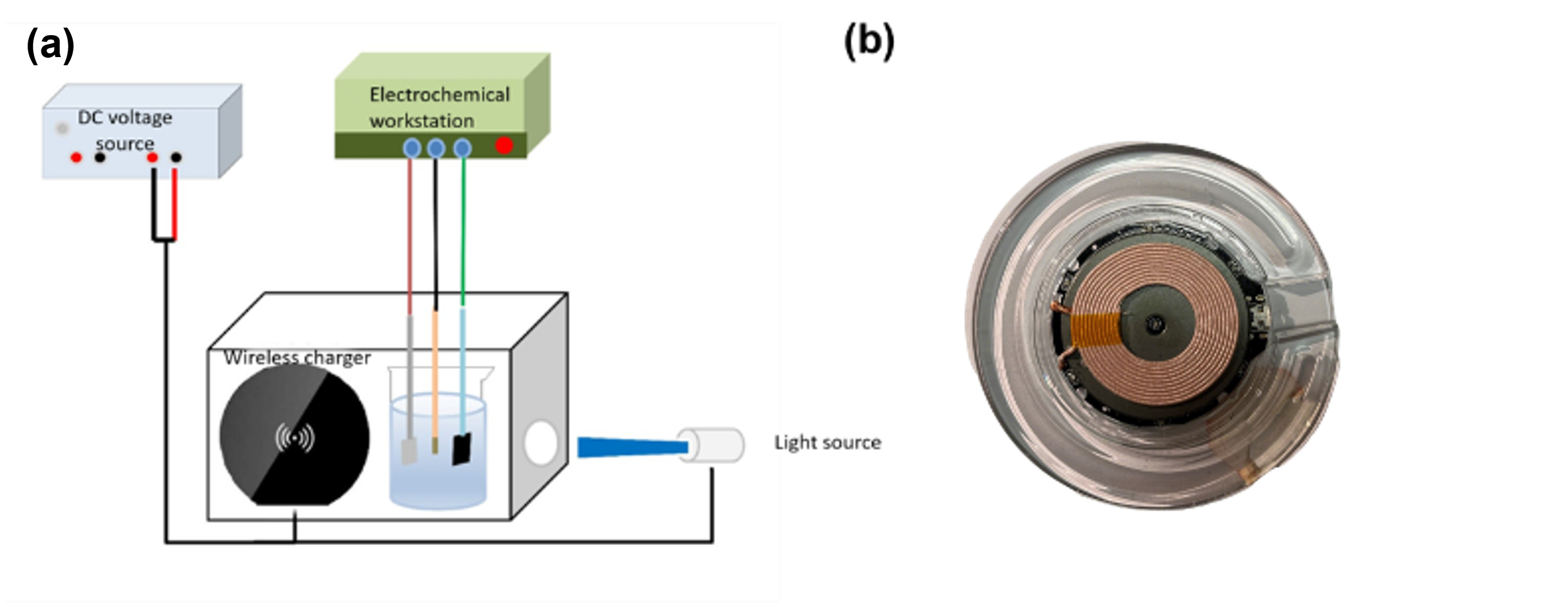


**Figure S8** (a) Schematic diagram of PEC and AEF performance testing, (b) internal structure of the wireless charger.

**Table S2 PEC performance comparison**

| Electrode | Transient current density | Wavelength of light | Power of light | References |
| --- | --- | --- | --- | --- |
| ZnO/BP | 0.6 μA cm^-2^ | 420 nm | 300 W | ^[24]^ |
| BiOCl/Ti_3_C_2_T_x_ | 150 nA cm^-2^ | —— | —— | ^[37]^ |
| BP/Ti_3_C_2_T_x_/TiO_2_ | 9 μA cm^-2^ | 355 nm | —— | ^[38]^ |
| BP/BiOCl | 2.5 μA cm^-2^ | 808 nm | —— | ^[45]^ |
| ZnO/Bi_2_O_3_/BiOCl/BP/MXene | 20.46 μA cm^-2^ | 405 nm | 30 W | Our work |

**Table S3 Comparison of coupling performance between PEC and AEF**

| Electrode | Current density under illumination | Coupled current density of AEF and light illumination | Coupling amplification factor | | References |
| --- | --- | --- | --- | --- | --- |
| WO_3_/Bi_10_O_6_S_9_/BP | 0.99 mA cm^−2^ | 1.59 mA cm^−2^ | | 1.6 | ^[10]^ |
| WO_3_/BiVO_4_/MXene | 1.15 mA cm^-2^ | 1.39 mA cm^-2^ | | 1.2 | ^[11]^ |
| ZnO/Bi_2_O_3_/BiOCl/BP/MXene | 20.46 μA cm^-2^ | 29 μA cm^-2^ | | 1.4 | Our work |

**Table S4 Comparison of coupling performance between PEC and AEF**

| Electrode | Cycling time | Cycling stability | References |
| --- | --- | --- | --- |
| WO_3_/Bi_10_O_6_S_9_/BP | 6000 s | 81.06% | ^[10]^ |
| borophene/bismuthene/MXene/BP | 8000 s | 84.61% | ^[12]^ |
| bismuthene/BP | 800s | 85.00% | ^[46]^ |
| ZnO/Bi_2_O_3_/BiOCl/BP/MXene | 10000 s | 84.86% | Our work |

**Table S5 Sensor parameters**

| Sensitivity | Resolution | Range |
| --- | --- | --- |
| 1.7 μA V^-1^ | 2.51*${10}^{-3}$ μA | -0.101~0.101 μA |

Sensitivity refers to the ratio of the change in output quantity to the change in input quantity when a sensor operates in a steady state, reflecting the degree of response of the sensor to changes in the measured quantity. Resolution refers to the smallest unit that a sensor can distinguish within its measurement range. Range refers to the range of values or signals that an instrument can measure. The resolution and range can be directly read from the test data of the electrochemical workstation.

$$Sensitivity=\frac{0.51 \mu A}{0.3 V}=1.7\mu A/V$$

**References**

1. Wu, Z.; Zhao, E.; Xiang, H.; Hao, X.; Liu, X.; Meng, J., Crystal structures and elastic properties of superhard IrN_2_ and IrN_3_ from first principles. *Physical Review* *B*. **2007**, *76*, 054115, https://doi.org/10.1103/PhysRevB.76.054115.

2. Mouhat, F.; Coudert, F, X., Necessary and sufficient elastic stability conditions in various crystal systems, *Phys. Rev. B.* **2014**, *90*, 224104, https://arxiv.org/pdf/1410.0065.
